# Language barriers in acute care in pediatric traumatology — Level 1 trauma center analysis

**DOI:** 10.1007/s00431-026-06909-3

**Published:** 2026-04-07

**Authors:** Vanessa Groß, Stephan Payr

**Affiliations:** 1 Department of Trauma Surgery, University Clinic of Orthopedics and Trauma Surgery, Medical University of Vienna, Waehringer Guertel 18-20, Vienna, 1090 Austria; 2 Section of Pediatric Trauma Surgery, Department of Trauma Surgery, University Clinic of Orthopedics and Trauma Surgery, Medical University of Vienna, Vienna, Austria

**Keywords:** Pediatric trauma, Language barriers, Acute care, Preverbal children

## Abstract

The growing linguistic diversity among children and their guardians is making language barriers an ever more critical issue in healthcare. Research shows that multiple facets of medical care can be impacted by language barriers. Yet, the potential influence of language barriers in pediatric traumatology remains largely unexplored. In this retrospective case–control study, the parameters adequacy of X-ray diagnostics, treatment duration, unscheduled revisits within 24 h and diagnostic corrections and/or additions were evaluated for a case group (with language barriers) and a control group (without language barriers). The observation period was 2014 to 2023. Language barriers were associated with lower odds of adequate X-ray diagnostics (OR = 0.53, 95% CI 0.30–0.94; *p* = 0.030), while the difference in treatment duration was small and not statistically significant (+ 2 min, 95% CI − 0.126–4.126; *p* = 0.066). Diagnostic corrections were more frequent in the language-barrier group (case: 3.0% vs. control: 0.4%; *p* = 0.045). Twenty-four-hour revisits were similarly low (case: 1.5% vs. control: 0.8%; *p* = 0.605).

*Conclusion*: Overall, language barriers did not obstruct the treatment process of children aged 0 to 3 years in a trauma care setting despite slight differences. These findings set traumatology apart from other medical fields, potentially due to the clearer visibility of injuries, the efficiency of non-verbal cues, and the focused nature of diagnostic procedures. Targeted communication support and even more standardized diagnostic pathways may help reduce variability.

**Table:** 

**What is Known:**	
• *Language-barrier effects have a wide range of effects on the diagnosis and treatment process for pediatric patients in various specialist areas.*	
**What is New:**	
• *Non-verbal communication forms and clearer injury patterns in traumatology help prevent diagnostic errors.*	
• *Language barriers do not clinically impact the treatment of preverbal children in pediatric traumatology.*

## Introduction

In medical settings, language barriers can become significant when patients and healthcare staff fail to communicate efficiently [1]. A lack of communication not only affects individual conversations between patients and medical providers, but it also impacts the entirety of patient care: from hospital admissions to discharges and single treatments [1–7]. With increasing rates of international migration [8], language barriers could turn into a growing issue of patient safety and quality of care. The international migration developments are also reflected on national levels [8–10]. Immigrant inhabitants often speak the language of their origin countries in their private households [10]. This increasing linguistic diversity is also noticeable among children, where about 27% predominantly use different languages [10].

In some cases, this can lead to poorer local language proficiency than in their native peers, since migrant children only acquire the local language through external social contexts, while they mainly use their parents’ heritage language in their daily lives [11]. Migrant children therefore may need more assistance in learning the local language.

Taking this into account, immigrant children with parents not fluent in the local country’s language may be especially vulnerable to patient safety hazards caused by language barriers [2–4, 7, 12, 13]. Due to the concept of infantile language development, the researched age group of 0 to 3-year-olds seems to be especially vulnerable.

At 3 years old, a normally developed child knows about 200 to 300 words, with a rudimentary understanding of syntax rules and basic grammar [14]. Now trying to speak to them in a different language than what they are used to at home may only complicate medical encounters.

Implications language barriers can have on the medical management of patients are limited access to medical treatment and complications during care tasks [2, 3, 13, 15, 16]. Furthermore, physicians may experience reduced confidence in choosing the right diagnostic procedures when assessing patients with language barriers — leading to a dependency on additional diagnostic tests [6, 17, 18]. For pediatric trauma care being this study’s setting, this is especially relevant due to the diagnostic usage of radiographic imaging and the resulting radiation exposure. Excessive diagnostic imaging may contribute to future health risks, e.g., triggering genetic changes over time [19]. Therefore, X-ray imaging must be applied with discretion rather than as a routine measure [19]. However, Zamor et al. have shown radiographic usage not in accordance with guidelines while diagnosing bronchiolitis in children with language barriers [20]. Further potential disadvantages can be prolonged waiting times, increased return rates to emergency departments, increased loss to follow-up, higher rates of non-adherence to prescribed treatments and increased potential for diagnostic errors and adverse events [2–5, 13, 18, 21–28].

Current research in medicine highlights the broad impact language barriers can have, particularly in relation to quality of care, patient safety and outcomes [2–4, 7, 12, 13]. However, most of the literature focuses on an adult patient collective rather than a pediatric cohort. Particularly effects on pediatric traumatology remain largely unaddressed in the current literature, despite children being especially vulnerable to possible adverse consequences as mentioned above.

The present study aims to address this research gap and examine the influence of language barriers on the traumatological treatment of preverbal children.

## Methods

The underlying study was carried out with the approval of the Ethics Committee of the Medical University of Vienna (16 January 2024; Code: 2058/2023) and according to the Declaration of Helsinki in its latest amendment. The study design was a retrospective case–control study. Data collection was carried out on the basis of the information management system (AKIM) of the studied hospital.

### Study population

All patients were treated at the Department of Trauma Surgery at the Medical University of Vienna in the timeframe of 2014 to 2023. The patients were between 0 and 3 years of age.

**Fig. 1:**
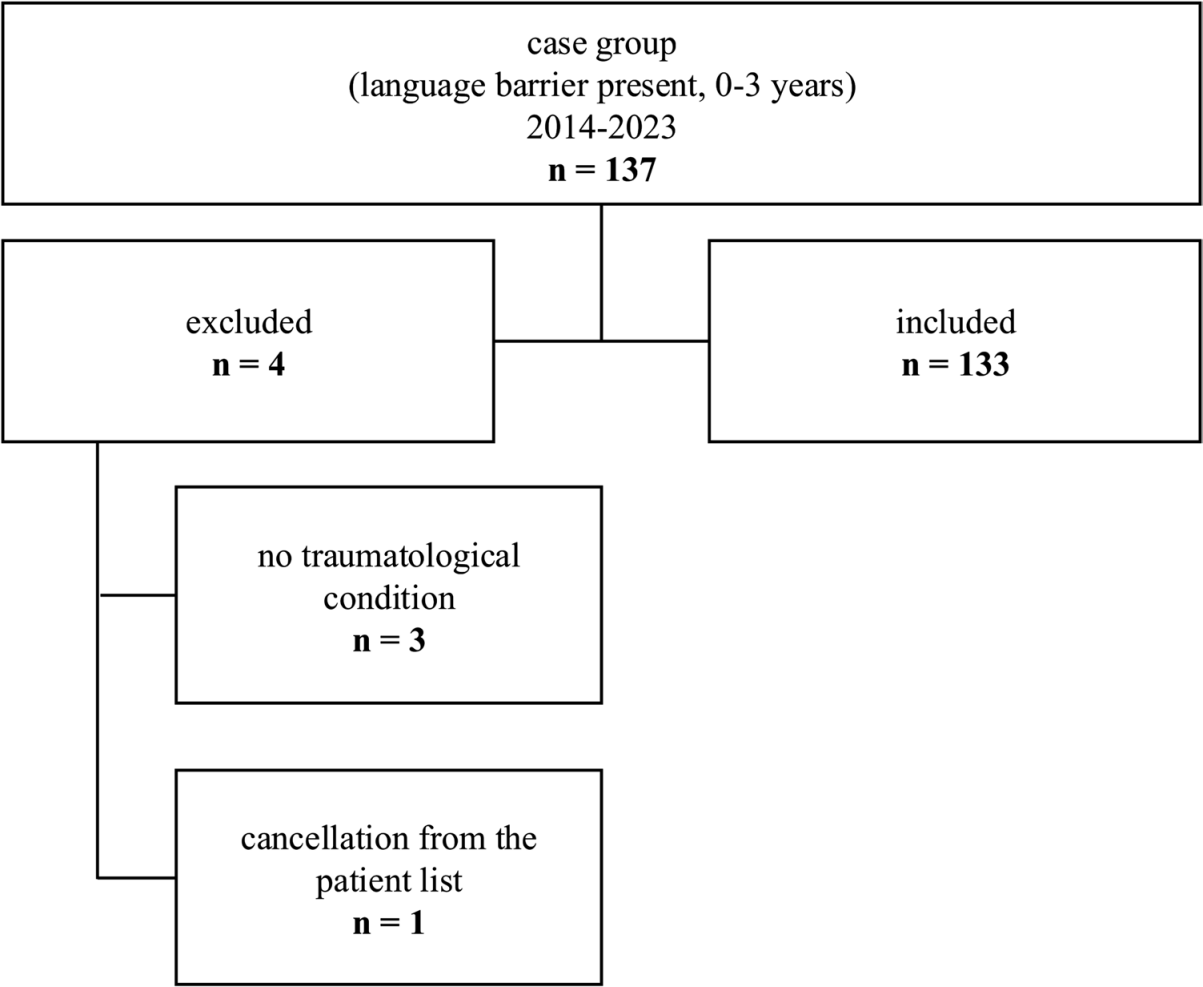
Flowchart of case group

**Fig. 2:**
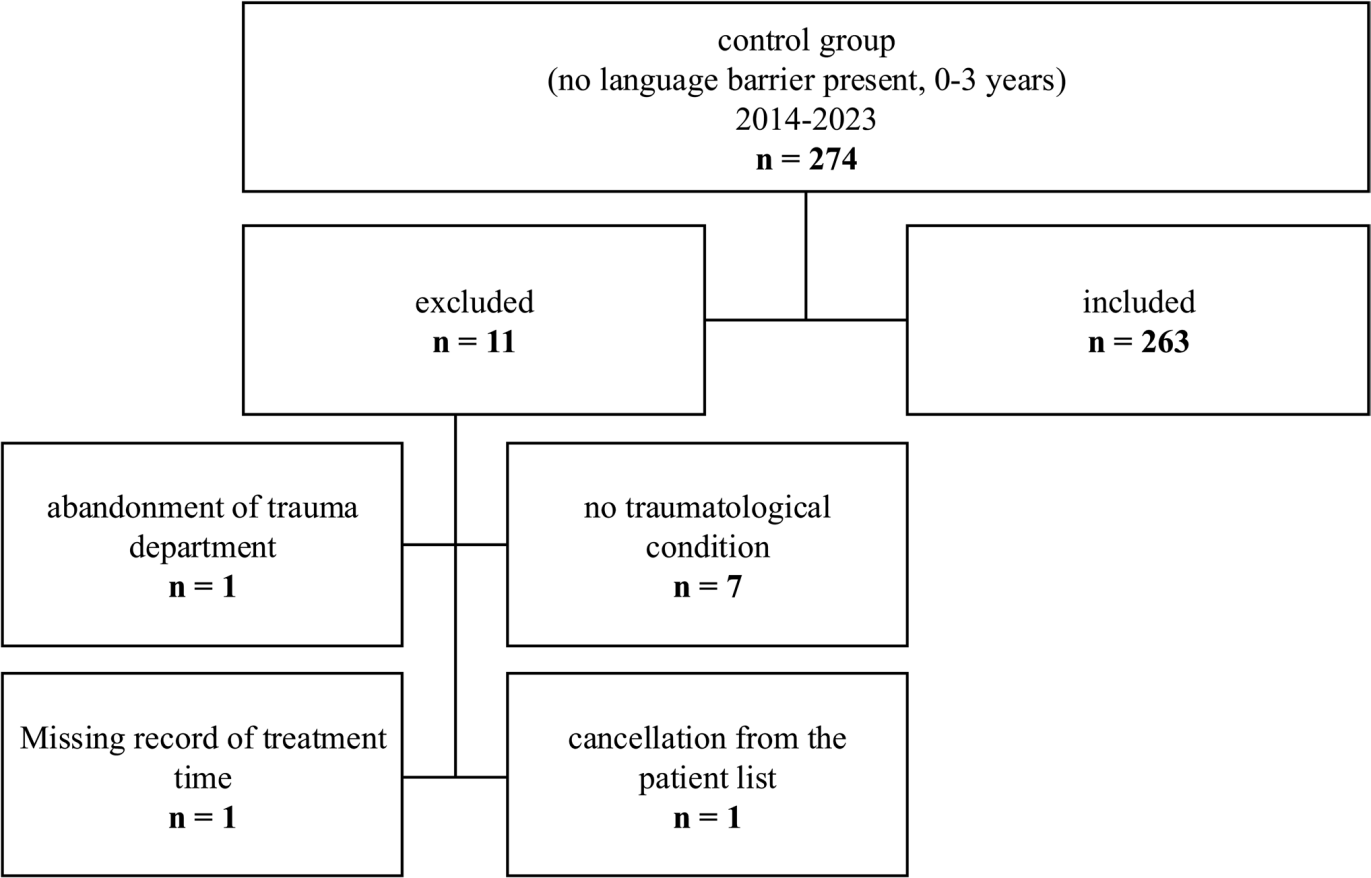
Flowchart of control group

Patients were assigned to the case group based on the documentation of the keyword “Sprachbarriere” (German for language barrier) in their accident records. One hundred thirty-seven eligible patients were identified, out of which 133 were included in this study (Fig. 1). The control cohort, which was twice the size of the case cohort, was matched by age and sex. Out of the initial 274 patients in the control group, 263 were included (Fig. 2).

### Data collection

For both cohorts, the same parameters were collected. Demographic data such as age and sex were identical in both groups due to the matching process. To get an overview on the examination and treatment process, variables such as the number of suspected injured regions, the number of radiologically investigated (X-ray) regions, and the type of treatment (conservative/surgical) were collected. Patients’ diagnoses were also collected. The principal diagnosis was defined as the first-listed diagnosis and collapsed into six clinically relevant categories and most frequently listed: (1) head contusion; (2) bodily contusion other than head; (3) laceration; (4) fracture/bone injury including (fracture, subperiosteal fracture, bone fissure, bone avulsion, and epiphysiolysis); (5) pulled elbow; and (6) other remaining diagnoses. Diagnostic complexity was defined as more than one diagnosis listed and treated as a secondary potential confounder. Furthermore, information on follow-up (FUP) processes was gathered. The investigated parameters included: scheduled FUP (in days), attendance to scheduled FUP, and symptoms at FUP (n/y).

This study’s primary objective was the comparison of 4 main parameters. These main parameters consisted of the following: adequacy of X-ray diagnostics, unscheduled revisits within 24 h, diagnostic corrections and/or additions, and the treatment duration.

The parameter adequacy of X-ray diagnostics was a novel and exploratory variable, specifically created for this study. It was evaluated as either adequate or inadequate. For this evaluation, the patient files were reviewed from the standpoint of the attending physician at the presentation. Later gathered information and test results were deliberately excluded to minimize hindsight bias. To still make consistent evaluation possible for all patients, the authors relied on predefined criteria as a guiding framework. These benchmarks included guidelines and standard practices at the investigated hospital. Experienced trauma surgeons were consulted in case of diagnostic uncertainties — reflecting standard clinical practice. Imaging was classified as inadequate when it deviated from the established benchmarks. This entailed both the overuse and unwarranted omission of radiographs.

Unscheduled revisits within 24 h recorded all patients who revisited the trauma department within a 24-h period following their initial registration.

Entries in the patient records explicitly marked as diagnostic errors were identified for the parameter diagnostic corrections and/or additions.

The parameter “treatment duration” was defined as the period during which patients received active care at their initial presentation. This variable was assessed using the AKIM-System. As patient treatment and documentation processes occur simultaneously, the active processing time of patient files was considered equivalent to the “treatment duration.”

### Statistical analysis

Patient characteristics were summarized descriptively by language-barrier group. All four study outcomes were first reported descriptively and compared between groups in unadjusted analyses. Categorical outcomes were compared using chi-square or Fisher’s exact tests, as appropriate, and treatment duration was compared using Mann–Whitney *U*-test with average age compared using independent *t*-test. Adjusted analyses were then performed for the two prespecified primary outcomes, adequacy of X-ray diagnostics and treatment duration. Adequacy of X-ray diagnostics was modeled using multivariable logistic regression, and treatment duration was modeled using multivariable median regression because of right skew. Both models included language-barrier group, matching factors of age and sex, and principal diagnosis category along with diagnostic complexity (multiple diagnoses: Y/N). Interactions between language-barrier group and each factor were tested in the adjusted models and retained when significant at the 0.05 level. Regression coefficients from the median regression model were accompanied by 95% confidence intervals obtained using bootstrap resampling, given the well-established use of bootstrap inference for quantile regression. Unscheduled revisits within 24 h and diagnostic corrections and/or additions were considered secondary exploratory outcomes because of sparse event counts and were reported only in unadjusted analyses.

Statistical evaluations were conducted with Microsoft Excel 16.90.2 and SPSS 29.0, with quantile regression conducted using R 4.4.2.

## Results

### Demography

After applying the inclusion criteria, the case cohort included 133 patients while the control cohort included 263 patients. Matched cohorts shared a similar age and sex distribution: mean age was 1.8 (± 1.0) years in both groups (Table 1); 42.7% of patients were female (case: 57, control: 112) while 57.3% were male (case: 76, control: 151).

**Table 1:** Characteristics of study population described overall and by case–control group. *p < 0.05, *p*-values were derived from the chi-square test for categorical variables (or Fisher’s exact test when expected cell counts were < 5) and from the independent-samples *t*-test for age and the Mann–Whitney *U* test for treatment duration (distribution was skewed)

*n* (%), mean ± SD, or median [IQR]	Overall (*N *= 396)	Control (*N* = 263) (w/out language barriers)	Case (*N* = 133) (with language barriers)	*p*-value
Age in years	1.8 ± 1.0	1.8 ± 1.0	1.8 ± 1.0	0.871
< 1 (infants)	34 (8.6%)	23 (8.7%)	11 (8.3%)	
1	127 (32.1%)	83 (31.6%)	44 (33.1%)	
2	107 (27.0%)	71 (27.0%)	36 (27.1%)	
3	128 (32.3%)	86 (32.7%)	42 (31.6%)	
Sex, male	227 (57.3%)	151 (57.4%)	76 (57.1%)	0.959
# Suspected injury regions, > 1	28 (7.1%)	14 (5.3%)	14 (10.5%)	0.056
# X-rays ordered				0.251
None	72 (18.2%)	52 (19.8%)	20 (15.0%)	
One	261 (65.9%)	174 (66.2%)	87 (65.4%)	
Two or more	63 (15.9%)	37 (14.1%)	26 (19.5%)	
# X-rayed regions				0.470
None	76 (19.2%)	51 (19.4%)	25 (18.8%)	
One	258 (65.2%)	175 (66.5%)	83 (62.4%)	
Two or more	62 (15.7%)	37 (14.1%)	25 (18.8%)	
Cranial x-ray, yes	191 (48.2%)	136 (51.7%)	55 (41.4%)	0.051
Type of Tx, surgical	7 (1.8%)	3 (1.1%)	4 (3.0%)	0.231
Attended FUP, yes	54 (13.6%)	26 (9.9%)	28 (21.1%)	0.002*
Primary diagnosis				0.118
Head contusion	110 (27.8%)	78 (29.7%)	32 (24.1%)	
Bodily contusion other than head	71 (17.9%)	50 (19.0%)	21 (15.8%)	
Laceration	87 (22.0%)	58 (22.1%)	29 (21.8%)	
Fracture/bone injury	53 (13.4%)	26 (9.9%)	27 (20.3%)	
Pulled elbow	27 (6.8%)	18 (6.8%)	9 (6.8%)	
Other	48 (12.1%)	33 (12.5%)	15 (11.3%)	
Multiple diagnoses, yes	96 (24.2%)	59 (22.4%)	37 (27.8%)	0.238
Outcomes				
Adequate X-ray diagnostics, yes	333 (84.1%)	228 (86.7%)	105 (78.9%)	0.047*
Unscheduled revisit w/in 24 h, yes	4 (1.0%)	2 (0.8%)	2 (1.5%)	0.605
Diagnostic corrections/adds, yes	5 (1.3%)	1 (0.4%)	4 (3.0%)	0.045*
Treatment duration in minutes	9 [6, 18]	8 [5, 16]	12 [6, 21]	0.003*

Since several patients had more than one diagnosis, a total of 178 diagnoses were collected in the case group, whereas the control group had a total number of 330 diagnoses. The primary diagnoses did not differ significantly between both groups (*p* = 0.118), although fracture/bone injury was more frequent among the case cohort (case: 20.3% vs. control: 9.9%). Multiple diagnoses were documented in 27.8% of patients in the case group and 22.4% of patients in the control group.

### Examination and treatment process

The proportion of patients with more than one suspected injury region was low but slightly higher in the case cohort (10.5%) compared to the control cohort (5.3%; *p* = 0.056). The number of X-rays ordered did not differ significantly between the cohorts (*p* = 0.251). Likewise, the number of X-rayed regions was comparable (*p* = 0.470). Cranial X-ray was performed in 41.4% of patients in the case cohort and 51.7% of patients in the control cohort (*p* = 0.051). Surgical interventions were rare, involving only 4 (3%) patients in the case group and 3 (1.1%) patients in the control group. Attendance to scheduled FUP was significantly higher in the case cohort compared to the control group (case: 21.1% vs. control: 9.9%; *p* = 0.002). However, the timing of FUP appointments was similar: the median was 3 days (mean: 4.07 ± 2.943) in the case group and 4 days (mean: 4.31 ± 3.172) in the control group.

### Main parameters

#### Adequacy

In unadjusted analyses, adequate X-ray diagnostics were observed less frequently in the case cohort than in the control cohort (case: 78.9% vs. control: 86.7%; *p* = 0.047) (Table 1). After adjustment for age, sex, primary diagnosis category, and multiple diagnoses, children with language barriers had 47% lower odds of adequate X-ray diagnostics than those without language barriers (OR = 0.53, 95% CI: 0.30–0.94; *p* = 0.030) (Table 2). Similarly, female children had 43% lower odds of adequate X-ray diagnostics than male children, with borderline statistical significance (OR = 0.57, 95% CI: 0.32–0.99; *p* = 0.050).

**Table 2:** Adjusted odds of receiving adequate X-ray diagnostics among children with language barriers compared with those without language barriers. *p*-value based on logistic regression in GLM model fully adjusted for all factors listed in table. Interaction effects between group and each covariate were evaluated and none were statistically significant (all *p* > 0.05); therefore, only main effects are presented

Outcome:	Adequate X-ray diagnostics	
	OR_ad_ (95% CI)	*p*-value
Language barriers		
No (controls)	Reference	
Yes (cases)	0.53 (0.30, 0.94)	0.030*
Age in years		0.903
< 1 (infants)	Reference	
1	1.09 (0.36, 3.34)	0.880
2	1.35 (0.42, 4.31)	0.618
3	1.32 (0.42, 4.17)	0.632
Sex		
Male	Reference	
Female	0.57 (0.32, 0.99)	0.50 ~
Primary diagnosis:		0.460
Head contusion	Reference	
Bodily contusion other than head	0.83 (0.33, 2.13)	0.704
Laceration	0.58 (0.25, 1.39)	0.223
Fracture/bone injury	1.29 (0.43, 3.85	0.650
Pulled elbow	0.47 (0.16, 1.42)	0.469
Other	0.58 (0.22, 1.54)	0.271
Multiple diagnoses		
No	Reference	
Yes	1.84 (0.83, 4.06)	0.134

#### Treatment duration

Treatment duration differed between groups in unadjusted analyses (case: median 12 [IQR 6, 21] min vs. control: 8 [IQR 5, 16]; *p* = 0.003). In adjusted analyses, children with language barriers had a slightly longer treatment duration than children in the control group (Table 3). The adjusted median treatment duration was 11.6 min in the case group compared to 9.6 min for the control group. This corresponded to an adjusted difference of 2min (95% CI: − 0.126–4.126; *p* = 0.066). Fracture/bone injury was associated with a significantly longer median treatment duration compared with head contusion (adjusted difference 13 min; 95% CI: 9.04–16.96; *p* < 0.001). All other diagnosis categories showed no significant differences in median treatment duration compared with head contusion (*p* > 0.05).

**Table 3:** Adjusted differences in median treatment duration (minutes) from the fully adjusted quantile regression model. *p*-value based on quantile regression (tau = 0.50, i.e., median) model fully adjusted for all factors listed in table; 95% CI were estimated using bootstrap resampling. Interaction effects between group and each covariate were evaluated and none were statistically significant (all *p* > 0.05); therefore, only main effects are presented

Outcome:	Treatment duration (minutes); median = 9 min		
	Average predicted median (95% CI)	Adjusted difference in median minutes (95% CI)	*p*-value
Language barriers			
No (controls)	9.6 (8.5, 10.9)	Reference	
Yes (cases)	11.6 (9.8, 13.3)	2 (− 0.126, 4.126)	0.066
Age in years			
< 1 (infants)	10.3 (7.5, 14.0)	Reference	
1	9.3 (8.0, 10.7)	− 1 (3.974, 1.974)	0.510
2	10.3 (8.5, 12.4)	0 (− 3.248, 3.248)	1.00
3	11.3 (9.6, 12.8)	1 (− 2.303, 4.303)	0.553
Sex			
Male	10.3 (9.2, 11.5)	Reference	
Female	10.3 (8.6, 12.0)	0 (− 1.957, 1.957)	1.00
Primary diagnosis:			
Head contusion	8.2 (6.7, 9.6)	Reference	
Bodily contusion other than head	9.2 (7.8, 11.0)	1 (− 1.027, 3.027)	0.334
Laceration	8.2 (6.8, 10.4)	0 (− 2.119, 2.119)	1.00
Fracture/bone injury	21.2 (16.4, 23.8)	13 (9.04, 16.96)	< 0.001
Pulled elbow	11.2 (8.4, 16.6)	3 (− 1.962, 7.962)	0.237
Other	8.2 (6.1, 9.9)	0 (− 2.189, 2.189)	1.00
Multiple diagnoses			
No	9.8 (8.7, 10.7)	Reference	
Yes	11.8 (9.5, 14.6)	2 (− 0.469, 4.469)	0.113

#### Revisit within 24 h

Within 24 h of their initial visit, 1.5% (2) of patients in the case cohort and 0.8% (2) of patients in the control cohort returned to the trauma department (*p* = 0.605). Due to sparse event counts, this outcome was evaluated only in unadjusted analyses.

#### Diagnostic corrections and/or additions

Diagnostic corrections or additions occurred in 3.0% (4) of the case group compared to 0.4% (1) in the control group (*p* = 0.045), indicating statistical significance. However, given the low number of events, this outcome was assessed only in unadjusted analyses.

## Discussion

Aligning with findings from prior research, the present study suggests that language barriers can affect care processes even in a high-volume Level 1 trauma setting. In unadjusted analyses, adequate X-ray diagnostics were less frequent in the language-barrier group than in controls. Importantly, this difference persisted after adjustment for age, sex, primary diagnosis and diagnostic complexity. Children with language barriers had 47% lower adjusted odds of receiving adequate X-ray diagnostics (OR = 0.53, 95% CI: 0.30–0.94; *p* = 0.030). Notably, the disparity in diagnostic adequacy does not seem to be explained by doing more X-rays overall. The number of ordered X-rays and X-rayed regions were similar between both groups, suggesting that the difference reflects imaging appropriateness rather than overall imaging volume. This, in turn, highlights the value of the new exploratory parameter “X-ray adequacy,” capturing both missed indications and unnecessary radiographs. These findings complement the mixed evidence on imaging utilization in language-barrier patients [18–20, 29]. Depending on the setting, studies have reported increased [18, 20], decreased [19], or unchanged imaging [29]. It is therefore plausible that differences in clinical environments, such as general pediatric emergency departments and trauma-focused departments, may shape imaging patterns [18–20, 29].

Regarding treatment duration, the results were more nuanced. For instance, prior work in pediatric emergency departments reported a 28-min longer stay in patients with language barriers [18]. Hampers et al. attributed this finding to more frequent hospital admissions, more intravenous therapies, and a greater number of diagnostic procedures [18]. In the present trauma setting, unadjusted treatment duration was longer among children with language barriers. However, in the adjusted model, the association between language barriers and treatment duration was small and did not reach conventional statistical significance (median case: 11.6 min vs. control: 9.6 min; difference 2 min, 95% CI: − 0.126–4.126; *p* = 0.066). This suggests that the unadjusted difference may be partly explained by differences in injury characteristics and diagnostic complexity rather than language barriers alone. More resource-intensive injury types such as fracture/bone injuries were strongly associated with longer treatment duration compared to head contusions, supporting the clinical plausibility of the model.

This study also assessed whether language barriers contribute to diagnostic errors, as impaired communication has been linked to treatment delays, prolonged recovery, and complications in other contexts [2, 6, 30]. For example, Stokes et al. suggested that children with appendicitis and language barriers were inadequately managed during their initial healthcare visits, due to critical illness indicators being “lost in translation” [28]. In the present study, diagnostic corrections and/or additions were secondary outcomes with sparse event counts and should therefore be interpreted cautiously. Nevertheless, they occurred more frequently among children with language barriers in unadjusted analyses (case: 3.0% vs. control: 0.4%; *p* = 0.045). This finding is consistent with previous reports of higher diagnostic errors among patients with language barriers in other medical fields [28, 31]. However, this pattern may be somewhat unexpected in pediatric trauma, where the clinical presentation is often more straightforward and supported by visible signs (e.g., bruising, open wounds, localized pain). Such presentations potentially reduce the reliance on verbal history. In line with this, Singh et al. reported that only 4% of pediatric diagnostic errors involved fractures [30]. For more reliable estimates, larger samples with higher event counts are required.

Furthermore, the existing literature indicates higher emergency department revisit rates among children (and parents) with language barriers [25, 32, 33]. Additionally, children aged 0 to 3 years are more likely to return to the emergency department, due to their limited speaking abilities, regardless of parental language barriers [34]. These findings did not align with the present study, where 24-h revisit rates were similarly low in both cohorts (case: 1.5% vs. control: 0.8%; *p* = 0.065). One possible explanation is a structured follow-up approach within the studied trauma department, due to heightened awareness among the staff. As a result, this may have reduced the need for unscheduled revisits.

Attendance to scheduled FUP was higher in the language-barrier cohort (case: 21.1% vs. control: 9.9%; *p* = 0.002). These observations should be interpreted cautiously since this may reflect surveillance or enrichment bias. Meaning, documented language-barrier cases may be skewed toward more serious injuries or presentations managed differently than less severe cases. Therefore, FUP data should be considered secondary outcomes in future studies rather than confounding variables.

Overall, the present findings suggest that language barriers in pediatric trauma are associated with a slightly lower diagnostic imaging adequacy and may be linked to small differences in care processes, while secondary outcomes require cautious interpretation due to sparse events. Together, these results underscore the importance of structured communication support and standardized diagnostic pathways to reduce variability in diagnostic decision-making for young children with limited ability to communicate symptoms.

Even though the study offers valuable new insights, certain limitations should be acknowledged. Its retrospective design may entail information loss, although documentation was complete for the variables analyzed. Despite the single-center study design, the study’s setting (Austria’s largest Level 1 trauma center) supports the representativeness of the patient population. The primary outcome models were adjusted for the slight imbalance in principal diagnosis, age, sex, and multiple diagnoses, but some residual confounding may still remain. Since the number of suspected injured body region (> 1) was borderline imbalanced between groups and rare overall (case: 10.5% vs. control: 5.3%), the usefulness for adjustment was limited. Similarly, the parameter cranial X-ray was not included as an adjustment variable since it rather reflected a separate diagnostic outcome than a true confounder. Higher FUP attendance in the language-barrier group may indicate surveillance or enrichment bias. In future studies, FUP data may therefore be treated as a secondary outcome. Additionally, the secondary endpoints in this study (24-h revisits and diagnostic corrections and/or additions) were rare and could only be analyzed in unadjusted comparisons. The parameter “adequacy of X-ray diagnostic” is an exploratory variable that is susceptible to observer bias. However, it has been introduced and has been found to be valuable.

## Conclusion

This study suggests that even in trauma care where visible injury signs and non-verbal communication may reduce reliance on verbal history, language barriers influence care processes. Overall, language barriers did not obstruct the treatment process of children aged 0 to 3 years in a trauma care setting despite slight differences. These findings set traumatology apart from other medical fields, potentially due to the clearer visibility of injuries, the efficiency of non-verbal cues, and the focused nature of diagnostic procedures. Targeted communication support and standardized workflows may help reduce variability in the future. Larger studies are needed to better assess secondary outcomes.

## Data Availability

All data generated and analyzed during this study are included in this published article. The datasets generated and analyzed during the current study are available from the corresponding author on reasonable request.
